# Multi-Omics Revealing the Response Patterns of Symbiotic Microorganisms and Host Metabolism in Scleractinian Coral *Pavona minuta* to Temperature Stresses

**DOI:** 10.3390/metabo12010018

**Published:** 2021-12-26

**Authors:** Jiayuan Liang, Wenwen Luo, Kefu Yu, Yongqian Xu, Jinni Chen, Chuanqi Deng, Ruiqi Ge, Hongfei Su, Wen Huang, Guanghua Wang

**Affiliations:** 1 Coral Reef Research Center of China, Guangxi University, Nanning 530004, China; jyliang@gxu.edu.cn (J.L.); shf2016@gxu.edu.cn (H.S.); wenhuang@gxu.edu.cn (W.H.); wgh@gxu.edu.cn (G.W.); 2 Guangxi Laboratory on the Study of Coral Reefs in the South China Sea, Nanning 530004, China; 3 School of Marine Sciences, Guangxi University, Nanning 530004, China; wenwenluo93@163.com (W.L.); 18127345798@163.com (Y.X.); chenjinnigx@163.com (J.C.); cqdeng163@163.com (C.D.); grace112237@163.com (R.G.); 4 Southern Marine Science and Engineering Guangdong Laboratory, Zhuhai 519080, China

**Keywords:** coral reefs, temperature stresses, symbiotic bacteria, dinoflagellates, metabolomics

## Abstract

Global climate change has resulted in large-scale coral reef decline worldwide, for which the ocean warming has paid more attention. Coral is a typical mutually beneficial symbiotic organism with diverse symbiotic microorganisms, which maintain the stability of physiological functions. This study compared the responses of symbiotic microorganisms and host metabolism in a common coral species, *Pavona minuta*, under indoor simulated thermal and cold temperatures. The results showed that abnormal temperature stresses had unfavorable impact on the phenotypes of corals, resulting in bleaching and color change. The compositions of symbiotic bacteria and dinoflagellate communities only presented tiny changes under temperature stresses. However, some rare symbiotic members have been showed to be significantly influenced by water temperatures. Finally, by using ultra-performance liquid chromatography tandem mass spectrometry (UPLC–MS) method, we found that different temperature stresses had very different impacts on the metabolism of coral holobiont. The thermal and cold stresses induced the decrease of anti-oxidation metabolites, several monogalactosyldiacylglycerols (MGDGs), and the increase of lipotoxic metabolite, 10-oxo-nonadecanoic acid, in the coral holobiont, respectively. Our study indicated the response patterns of symbiotic microorganisms and host metabolism in coral to the thermal and cold stresses, providing theoretical data for the adaptation and evolution of coral to a different climate in the future.

## 1. Introduction

The coral reefs play a vital role in maintaining the biodiversity and ecosystem functions of marine, and global climate change threatens them all over the world [1]. Coral is a temperature sensitive marine organism, which bleaches and dyes under abnormal sea temperatures [2]. As the main consequence of climate change, ocean warming is seriously threatening the survival of coral reefs [3]. Many studies have been conducted to explore the response patterns of corals to the elevated temperature by diverse methods [4,5,6]. For example, divergent responses to heat stress by different coral taxa has been found in the Great Barrier Reef, resulting in the regional-scale shift in the composition of coral assemblages [7]. Another study also conducted on the Great Barrier Reef revealed that the populations of corals successfully adapted the warming in the future but with increasing sensitivity to random thermal fluctuations [8]. Compared with the well-known warming, climate change will also lead to extreme weather events, such as abnormal cold in winter in tropical or subtropical regions. The abnormal temperature drop also can affect the growth and stability of coral reefs. However, there is a lack of research on the response patterns of coral reef to the relatively cold seawater temperature.

A variety of microorganism mutuality symbiosis with the corals and their interaction is indispensable in the normal physiological function of coral reefs [9,10]. Particularly, coral bleaching of coral reefs under the abnormal temperatures is the result of dysfunction of the symbiotic relationship and expulsion of the symbionts from coral host [11]. Among these symbionts, bacteria and dinoflagellates are more worthy of attention. Corals provided ideal habitats for bacteria, and numerous novel bacteria have been detected in them to complete substantial functions linked with nutrient cycling, the degradation of pollutants, and host health [12,13,14]. The dinoflagellates, mainly Symbiodiniaceae, is an obligate symbiotic family residing within the tissues of corals, which has been demonstrated in a particular relationship with coral resilience to ambient temperatures [15]. There is a two-way exchange of metabolites between these symbionts and the coral hosts to maintain a healthy state of mutualism [16] Changes in water temperature conditions may shift the symbiotic microbial communities of corals and destroy of the homeostasis of holobiont metabolism. Therefore, understanding the response patterns of symbiotic microorganisms and host metabolism in corals under temperature stresses is necessary to improve the formulation of coral reef conservation.

Recent advances in the omics-based technologies have provided more comprehensive methods to obtain the symbiotic microbial communities and metabolic changes in corals. High-throughput sequencing based on target biomarkers, such as 16S rRNA and ITS genes, has been frequently used to assess the diversity and composition of bacteria and dinoflagellates among diverse ecosystems, including the coral holobiont [17,18]. Moreover, metabolomics based on mass spectrometry have applied to explore the variations of metabolic functions in corals with different physiological states [19]. In this study, a multi-omics method combined with the high-throughput sequencing based on 16S rRNA and ITS genes and the un-targeted mass spectrometry-based metabolomics was established. *Pavona minuta*, a typical massive scleractinian coral from Weizhou Island, Beibu Gulf of China, was selected as the research object. The response patterns of the symbiotic microorganisms and host metabolism as well as the relationships among them under extremely high and low seawater temperature stresses were evaluated. The findings of this study will assist future research into the mechanisms of coral devastation under different environments and climate change.

## 2. Results

### 2.1. Changes in the Phenotypes of P. minuta under Temperature Stresses

**Figure 1:**
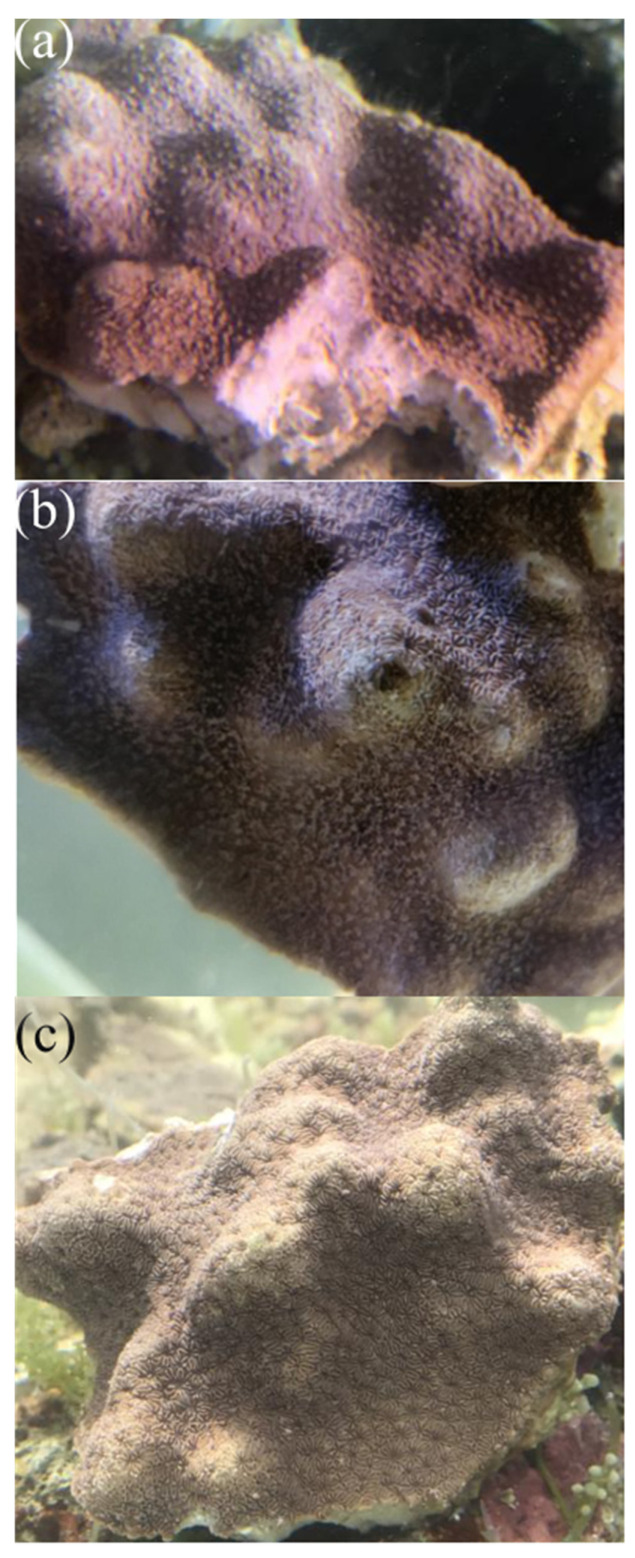
Changes in the phenotypes of corals under different temperature stresses. (**a**) 18 °C, LT; (**b**) 26 °C, CK; and (**c**) 34 °C, HT.

By applying a sustained increase stimulation to the coral cultured in our lab, the changes in phenotypes of *P. minuta* under extreme high and low water temperatures were observed. Obviously coral bleaching was seen in the corals from HT group, and the color of *P. minuta* turned from brown to red in the LT group (Figure 1). These resulted demonstrated the unfavorable impacts of abnormal water temperature on the corals.

### 2.2. Responses of Symbiotic Bacteria under Temperature Stresses

**Figure 2:**
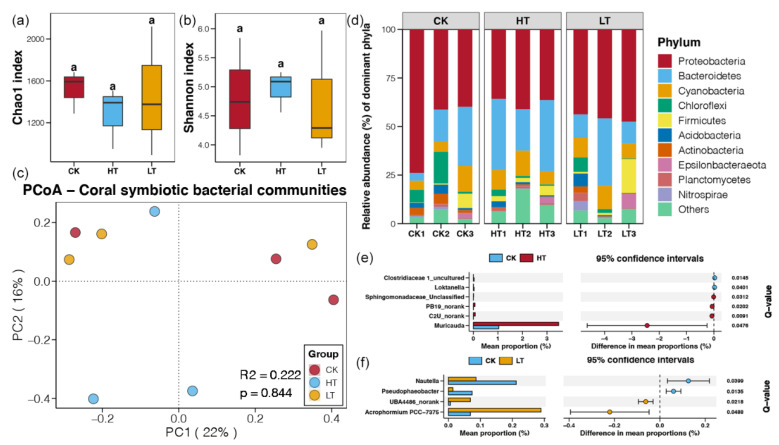
Responses of symbiotic bacterial communities under different temperature stresses. (**a**) Differences of Chao1 index among different groups. (**b**) Differences of Shannon index among different groups. Different lowercases letters above each box in the same subfigure represent significant differences between groups (Student’s *t*-test, *p* < 0.05). (**c**) PCoA and PERMANOVA of the symbiotic bacterial communities of corals under different temperature stresses. (**d**) The relative abundances of dominant phyla in the symbiotic bacterial communities of corals under different temperature stresses. (**e**) Bacteria with significantly different abundances in between corals from the HT and CK groups. (**f**) Bacteria with significantly different abundances in between corals from the LT and CK groups.

In this study, the symbiotic bacterial communities of *P. minuta* under different temperature stresses were investigated by Illumina sequencing based on 16S rRNA genes. A total of 482,518 high quality tags were obtained from nine samples, which were clustered into 3271 bacterial OTUs. These detected bacteria were classified into 45 phyla, 99 classes, 229 orders, 385 families, and 743 genera. The community complexity of symbiotic bacteria was evaluated using alpha-diversity based on data at the OTU level, including the OTU richness (Chao1) and the Shannon indices. However, no significant differences of Chao1 and Shannon indices were found among the bacterial communities of different groups (*p* > 0.05, Figure 2a,b). Moreover, the results of PCoA and PERMANOVA also presented similar composition of bacterial communities in corals under different temperature stresses (*p* > 0.05, Figure 2c). These results suggested that the temperatures stresses could not have a significant effect on the diversity and composition of symbiotic bacteria community in coral reefs.

The majority of bacterial OTUs in all samples belonged to the phyla of Proteobacteria (45.05%), followed by Bacteroidetes (22.63%), Cyanobacteria (9.20%), Chloroflexi (4.30%), and Firmicutes (4.14%). Although the relative abundance of different bacterial phyla was changed among different samples, while no significant difference was uncovered (*p* > 0.05). This result was agreement to the findings above (Figure 2a–c) and further indicated the limited influence of temperature stresses on the symbiotic bacteria community in coral reefs. Even so, we still found that a small number of bacterial genera changed significantly in abundance under temperature and pressure conditions. In HT samples, the genera of *Muricauda*, C2U_*norank*, PB19_*norank*, and *Sphingomonadaceae*_*Unclassified* were significantly enriched, while the relative abundances of *Loktanella* and *Clostridiaceae* 1_uncultured were significantly decreased (*p* < 0.05, Figure 2e). In TL samples, the genera of *Acrophormium* PCC-7375 and UBA4486_norank were more abundant, while the relative abundances of *Pseudophaeobacter* and *Nautella* were significantly declined (*p* < 0.05, Figure 2f).

### 2.3. Responses of Symbiotic Dinoflagellate Communities under Different Temperature Stresses

**Figure 3:**
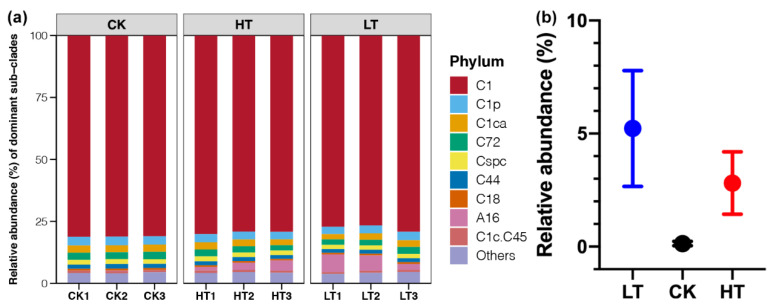
(**a**) The relative abundance of dominant dinoflagellate sub-clades across different samples. (**b**) Differences of the relative abundances of sub-clade A16 among different groups.

The relative abundance of dominant dinoflagellate is given in Figure 3a. At the clade level, Cladocopium (Clade C) was the dominant in all samples, which accounted for 89–96% in abundance of each sample. Symbiodinium (Clade A) was the second most abundant clade, which accounted for no more than 8% across all samples. At the subdivision level, C1 was overwhelmingly dominated in all samples with the 77–81% of the total communities, followed by C1p, C1ca, C72, Cspc, C44, and C18 (Figure 3a). There were few changes in the relative abundance of Clade C or sub-clades of it across samples under different temperature stresses (*p* > 0.05). In contrast, sub-clade A16 was the dominant member detected in Clade A, which had significantly higher abundances in sample from the HT and LT groups compared to those from the CK group (*p* < 0.05, Figure 3b).

### 2.4. Responses of Metabolome of Corals under Different Temperature Stresses

**Figure 4:**
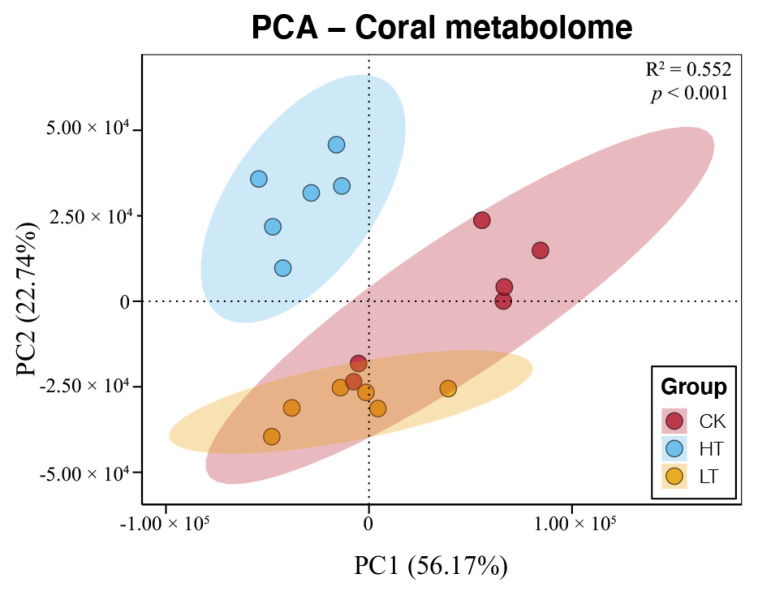
PCA and PERMANOVA of the metabolome of corals under different temperature stresses.

**Figure 5:**
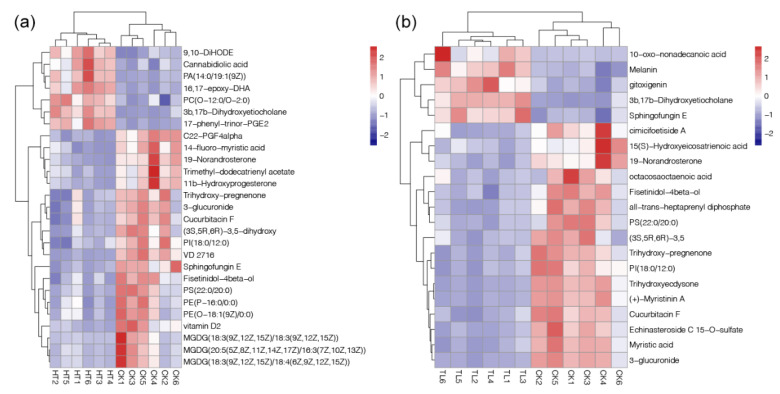
Heatmaps of the DAMs identified from the comparisons of HT-vs-CK (**a**) and LT-vs-CK (**b**).

According to the matching of mass spectrometry fingerprint and liquid chromatography retention time, the total amount of metabolites was identified and quantified. A total of 14,755 peaks were detected by UPLC-MS, and 1984 metabolites were annotated. PCA results showed the separated clustering patterns of metabolome from samples under different temperature stresses, suggesting significant variations of coral metabolism (PERMANVOA, *p* < 0.05, Figure 4). Moreover, 27 and 21 metabolites were identified as the DAMs in the comparisons of HT-vs-CK and LT-vs-CK, respectively (Table 1). Among them, the down-regulated DAMs were more abundant with 20 and 16 in HT-vs-CK and LT-vs-CK, respectively. In contrast, the up-regulated DAMs were only 7 and 6 (Table 1). The fold changes of identified DAMs were shown in heatmaps of Figure 5. The results showed that increased temperature had apparent effects on organic acids, amino acids, and fatty acids (Figure 5a). In contrast, decreased temperature had a very different impact on the metabolism characteristics in corals compared to the heating treatment, which mainly affected the organic acids (Figure 5b).

## 3. Discussion

Coral is a typical mutually beneficial symbiotic organism (together termed the coral holobiont), and the compositions coordinate with one another when coping with environmental stress. Therefore, a synthesized understanding of the multiple responses of the coral holobiont (including bacteria and dinoflagellate) to temperature is favorable for a deeper understanding of the ecological and evolutionary mechanisms of coral responses to climate change. Many previous studies have reported the responses of coral-microbe assemblages to extreme thermal stress, and showed inconsistent results of resistance or susceptibility of coral communities to climate change [20,21,22]. The data present herein illustrated the response patterns of symbiotic microorganisms and host metabolism in coral *P. minuta* exposed to different temperature stresses.

The fitness of coral holobiont has been proven to have an essential relationship with coral-associated bacterial communities [23]. Bourne et al. [24] demonstrated conserved bacterial banding profiles during a bleaching event by denaturing gradient gel electrophoresis analysis. The development of next-generation sequencing has been facilitating the research on diversity and community structure of microorganisms, and more compete symbiotic bacterial communities of corals have been detected by recent studies [14,18]. Proteobacteria, Bacteroidetes, Cyanobacteria, Chloroflexi and Firmicutes were found to be the dominant bacteria phyla in this study (Figure 2d), and this result was consistent with other studies [14,25]. For dinoflagellate, it is an obligate symbiotic member residing within the tissues of coral reefs, and most coral–dinoflagellate associations have host specificity [26]. As the advances of next-generation sequencing, the IST2 rDNA genotyping based sequencing were more frequently used to assess dinoflagellate diversity to obtain more comprehensive results [17,27]. The dinoflagellate community primarily consisted of Clade C (Cladocopium), in which C1 was the dominant symbiont (Figure 3). Other research about corals in marine areas, such as Okinawa of Japan and Jeju Island of Korea, were also found sub-clade of C1 as the majority member of the dinoflagellate–coral associates [28,29].

In contrast to the dominant symbiotic microorganisms, some rare members in symbiotic communities observed significant changes under different temperature stresses (Figure 2e,f, and Figure 3). In marine ecosystems, rare members of bacterial com-munities have been indicated to play a more active role than the dominant members [30]. Besides, rare members of dinoflagellate have long been recognized to contribute to the resilience of coral–algal associations [22]. Members of dinoflagellate subclades (e.g., D1a and A3) with relatively lower abundances in corals were known to be related to the thermal tolerance [31]. Several rare bacterial genera, including *Muricauda*, *Loktanella*, *Acrophorminum*, *Pseudophaeobacter*, and Hautella, were found to be significantly respond to the temperature stresses (Figure 2d,e). Among them, *Muricuada* had a relative abundance below to 3% and other genera were all below to 0.3%. The genus of *Muricuada* have been reported to possess the capacity of zeaxanthin biosynthesis [32], which is a natural pigment with critical role in the prevention of age-related macular degeneration in human beings [33]. The significant enrichment of *Muricuada in* corals from HT group indicated that this bacteria genus could respond to the tolerance of high temperature (Figure 2d). In addition, the bacteria genera that depleted in the HT or LT groups, such as *Loktanella* and *Pseudophaeobacter*, were showed to have the algicidal activity on the toxic dinoflagellate [34] or probiotic effect against to pathogens [35]. These results suggested the temperature stress on corals could cause the enrichment of harmful symbionts due to the weakness of competition. Moreover, we observed that Clade A16 of dinoflagellate experienced significant fluctuation in the results of this study, although its proportion in the dinoflagellate community of *P. minuta* was low (Figure 3b). According to the information mentioned above, we inferred that the sub-clade A16 of dinoflagellate could be inferred to have toxic on coral reefs, but the related evidence has not been reported in any previous studies.

Temperature stress can result in the imbalance of metabolism in the coral holobiont. Hillyer et al. [36] studied the metabolite profiles of symbiont and host during heat-stress and bleaching in a model cnidarian-dinoflagellate symbiosis, and detected elevated pools of polyunsaturated fatty acids (PUFAs) in the symbiont, but reductions of PUFAs in the host. Moreover, another study reported that elevated ambient temperatures could induce the alteration of carbohydrate composition, cell structural lipids, and signaling com-pounds in the reef-building coral [37]. In this study, a significant increase in the contents of some PUFAs, such as PA (14:0/19:1(9Z)), 16,17-epoxy-DHA, PC (0-12:0/0-2:0), and cannabidiolic acid, were also found in corals under thermal stress (Figure 5a). Moreover, the most decreased metabolites in corals under thermal stress were serval members of MGDG (Figure 5a). MGDG has been shown to have anti-oxidation ability, which can clear excess active oxygen free radicals in the hosts [38]. For cold stress, 10-oxo-nonadecanoic acid was the metabolites with the highest abundant increase (~20-fold increase, below 5-fold for other DAMs, Figure 5b), which is an inducer of lipotoxic effects and result in apoptosis [39]. These findings indicated the harmful effects of temperature stresses on the coral reefs through the changed of metabolic profiles.

In our study, almost all dominant bacteria and dinoflagellates seemed to have tiny variations under the temperature stress (Figure 2d and Figure 3). There is a theory called “limited symbiont shuffling”, which indicates that the composition of symbionts may change over time with their hosts simultaneously, but the change proportion of most of symbiotic members are relatively low and the occurrence of symbiont shuffling may be rare [26]. In this study, our results emphasized that the relative abundances of different clades kept relatively stable, which is consistent with limited symbiont shuffling theory [7,40]. The symbiotic microbial community studied in here presented a rich diversity (Figure 2 and Figure 3). This high diversity might hamper the predictions about their responses to simulated temperature stresses [41]. A useful method is to classify microbial communities into different functional groups instead of detecting the complicated responses of hundreds of microbial taxa. Furthermore, due to the heterogeneity of the sensitivity of different lineages, it would be intended to focus on these key species or their relatives in this kind of research. Future research may take a phylogenetic, trait-based framework involved in predicting coral-associated microbial responses to climate change. More indicators, such as trait diversity, should be introduced to the study of coral holobiont.

## 4. Materials and Methods

### 4.1. Experimental Specimens

Three colonies of coral *P. minuta* were collected by SCUBA diving using hammer and chisel at a depth of 4–6 m from the Weizhou Island (109°06′40′′ E, 21°04′30′′ N), Beibu Gulf in the South China Sea. Each colony was about 20 × 20 cm in size. All collected colonies were transported to the laboratory within 24 h to ensure their activity. Each colony was cut into 5 × 5 cm fragment samples and randomly placed in three coral incubation tanks (10 samples in each tank) for adaptive domestication at 26 °C for one month. As the optimal temperature for reef-building corals is 25–29 °C, here the temperature of 26 °C was chosen as the domestication temperature. The size of the aquaculture tank is 600 × 600 × 600 mm (length × width × height). The water flow speed was 6500 L·h^−1^. The environmental parameters of the water were as follows: 30–35‰ salinity, pH 7.3–8.3, 380–450 ppm Ca^2+^ concentration, 1200–1320 ppm Mg^2+^ concentration. The water movement in each tank was provided by small submersible pumps (CP-55, Zhongshan Jiebao Electronic Appliance Co., Ltd., Zhongshan, China). The light was set for a 12-h light-dark cycle using T5HO lights (Zhongshan Songbao Electronic Appliance Co., Ltd., Zhongshan, China).

### 4.2. Stimulation Experiments of Temperature Stresses

The sustained increase experiment method was used in this study, in which coral samples were exposed to a static temperature, not a periodic temperature change. After the domestication, a heating and cooling experiment was carried on with the samples in the three tanks. The water temperature in each tank was controlled by a temperature-controlled system (HS-66A, Guangdong Haili Group Co. Ltd., Chaozhou, China). One of the tanks was set as the control (CK), and all the environmental conditions were kept the same as the domestication (water temperature remained at 26 °C). The other two tanks were manipulated for heating and cooling, respectively, and the other environmental parameters of the water were kept unchanged except for water temperature. For the heating group (high temperature, HT), the water temperature was increased at the rate of 1 °C per day in a succession of eight days. The samples in the heating tank were cultured for three days after the temperature was raised to 34 °C. For the cooling group (low temperature, LT), the temperature was decreased at the rate of 1 °C per day to 18 °C and kept for three days. The choice of experimental temperatures was according to a pre-experiment, which indicated 34 °C and 18 °C as the highest and lowest temperature for coral surviving, respectively. After that, three coral samples from three randomly selected colonies as three replicates in each tank (about 50 mg for each sample) were collected. These samples were placed into liquid nitrogen instantly when taken from the tank, and then stored at −80 °C for the DNA sequencing and metabolomic analysis.

### 4.3. DNA Extraction and Sequencing

The total DNA of corals were extracted using the TIANamp Marine Animal DNA Kit (DP324, TIANGEN BIOTECH CO., LTD., Beijing, China) according to the manufacturer’s instructions. Agarose gel of 1.5% concentration was used to detect whether DNA was successfully extracted through electrophoresis. Then, the concentrations and purity of each successfully extracted DNA sample were measured by NanoPhotometer Classic Launched (IMPLEN, Munich, Germany). All DNA samples were stored at −20 °C until further use.

To obtain the bacterial and dinoflagellate communities in the corals, the V3-V4 region of 16S rRNA gene and the ITS gene in each extracted DNA were amplified by PCR using 338F-806R (338F: 5′-ACT CCT ACG GGA GGC AGC AG-3′, 806R: 5′-GGA CTA CHV GGG TWT CTA AT-3′) [42] and ITSintfor2-ITSreverse (ITSintfor2: 5′-GAT TGC AGA ACT CCG TG-3′, ITSreverse: 5′-GGG ATC CAT ATG CTT AAG TTC AGC GGG T-3′) [17], respectively. PCR reactions were performed in triplicates, with a 20 μL mixture containing 4 μL of 5 × FastPfu Buffer, 2 μL of 2.5 mM dNTPs, 0.8 μL of each primer (5 µM), 0.4 μL of FastPfu Polymerase, and 10 ng of the template DNA. The reaction conditions of PCR were as follows: 95 °C for 3 min, followed by 27 cycles at 95 °C for 30 s, 55 °C for 30 s, 72 °C for 45 s, and a final extension at 72 °C for 10 min. Amplicons were extracted from 2% agarose gels and purified using the AxyPrep DNA Gel Extraction Kit (Axygen Biosciences, Union City, CA, USA), following the manufacturer’s instructions. The extracts were then quantified using QuantiFluorTM-ST (Promega, Madison, WI, USA). Purified amplicons were pooled at equimolar concentrations and pair-end sequenced on an Illumina MiSeq PE300 platform (Shanghai Majorbio Biopharm Technology Co., Ltd., Shanghai, China). The raw reads for all samples are depositing into the NCBI Sequence Read Archive database with accessible number SRP314147.

### 4.4. Data Processing of Microbial Communities

Raw pair-end reads were merged using FLASH [43] after filtering adaptor sequences and removing low-quality reads to generate clean tags [22]. These tags were then assigned to operational taxonomic units (OTUs) using the UCLUST algorithm [44] at the 97% identity by QIIME v1.9.0 [45] The representative sequences of each OTU were selected as the tags with the highest numbers. For 16S OTU, the representative sequences were appointed to a taxonomy based on the SILVA 138 database [46]. For ITS OTU, the representative sequence to be aligned to the ITS2 database using BLASTn [21], and non-Symbiodiniaceae OTUs were removed [27]. Then, the bacterial and dinoflagellate OTU abundance tables were normalized using a standard number of tags according to the sample with the least number of tags.

### 4.5. Metabolites Extraction and UPLC-MS Analysis

For each sample, coral materials were ground into powder in liquid nitrogen, and 100 mg coral powder was transferred to a 1.5 mL Eppendorf tube. 600 μL of cold methanol/water solution (*v*:*v* = 4:1), and 20 μL of 2-chloro-L-phenylalanine (0.3 mg/mL) dissolved in methanol as internal standards were added to each sample. Two small steel balls were added to the tube and sonicated at 60 HZ for 2 min. After, the samples were ultrasonicated in the ice bath for 30 min and then sat for 20 min. The samples were centrifuged at 10,000 rpm for 10 min at 4 °C. Meanwhile, quality control (QC) samples were prepared by combining equal aliquots of replicates from each sample. During the instrument analysis, a QC sample was inserted every 3 coral samples to monitor the repeatability of the analysis process.

The samples were analyzed on a Waters UPLC I-class system equipped with a binary solvent delivery manager and a sample manager, coupled with a Waters VION IMS Q-TOF Mass Spectrometer equipped with an electrospray interface (Waters Corporation, Milford, CT, USA). A BEH C18 column (100 mm × 2.1 mm, 1.7 μm; Waters, Milford, CT, USA) was utilized to separate the metabolites with the column oven at 45 °C. The mobile phase consisted of solvent A (water containing 0.1% methanoic acid) and solvent B (acetonitrile containing 0.1% methanoic acid). The gradient elution procedure was set as follows: 0–2 min: 5–20% phase B; 2–8 min: 20–60% phase B; 8–12 min: 60–100% phase B; 100% phase B for 2 min; 14–14.5 min: 100 % to 5% phase B; 5% phase B for 1 min. The flow rate was 0.40 mL/min, and the injection volume was 3 μL. The MS conditions were as follows: electrospray capillary voltage: 1.0 kV; sampling voltage: 15 V; collision energy (CE): 6 eV; mass range: 50–1000 *m*/*z*; carrier gas flow: 900 L/h; scanning time and interval time: 0.1 s and 0.02, respectively. The ion source temperature and solvent removal temperature are respectively 120 °C and 500 °C. The signal was collected by positive and negative ion scanning mode. For metabolite identification, MarkerView software (DH Tech. Dev. Pte. Ltd., Singapore) was used to draw the peak information from the raw data to obtain the characteristics of metabolites, including *m*/*z*, retention time, and ion area. The annotations of metabolites were performed using the Human Metabolome Database (HMDB) [47].

### 4.6. Statistical Analyses

Alpha diversity indices of the bacterial communities, including Chao1 and Shannon indices, were calculated using the “vegan” package in R v4.0.2. Differences in these alpha diversity indices between HT and LT groups compared to the CK group were analyzed using the Student’s *t*-test. Variations in the bacterial community compositions of different samples were evaluated by principal coordinate analysis (PCoA) and PERmutational multivariate analysis of variance (PERMANOVA) based on the Bray–Curtis distance by the “vegan” packages in R v4.0.2. A stacked bar graph was used to show the relative abundance of dominant bacterial phyla and dinoflagellate subclades in different samples. Bacteria and dinoflagellate with different relative abundances between the temperature stressed and normal corals were recognized by the Student’s t-test using the STAMP software [48]. MetaboAnalyst v3.0 [49] was used to normalize the metabolite data for subsequent statistical analyses. Unsupervised principal component analyses (PCA) was performed on LC-MS data to compare the variations of metabolome of coral under different temperature stresses. The variable importance in projection (VIP) value, Student’s *t*-test, and fold change were used to recognize the differentially abundant metabolites (DAMs) as the intersection of the following criteria: (1) VIP value ≥ 1.5; (2) fold change ≥ 1.80 or ≤ 0.56; and (3) *p*-value < 0.05. The content distribution of DAMs in different samples was exhibited by the “pheatmap” package in R v4.0.2.

## 5. Conclusions

Our work exhibited the short-term response patterns of coral–microbe assemblages to abnormal temperatures from various perspectives. The diversity and composition of symbiotic bacterial communities was not found to be significantly affected by the temperature stresses. Meanwhile, the dominant dinoflagellates were also not significantly changed. However, some rare symbiotic bacterial genera and dinoflagellate sub-clade were found to be significantly influenced by the temperature stresses. Among them, the potential probiotic members were significantly depleted in the coral holobiont under both thermal and cold stresses. Moreover, many metabolites were observed with the significantly different abundances between the corals at normal condition and temperature stresses. The thermal and cold stresses induced the decrease of anti-oxidation and the increase of lipotoxic metabolites in the coral holobiont, respectively. This work provided insight into the responses of symbiotic microorganisms and physiological metabolisms involved in coral *P. minuta* under temperature stresses.

## Figures and Tables

**Table 1:** The numbers differentially abundant metabolites in corals under different temperature stresses.

Comparisons	Up-Regulated	Down-Regulated
HT-vs-CK	7	20
LT-vs-CK	6	15

## Data Availability

The raw reads for all samples are depositing into the NCBI Sequence Read Archive database with accessible number SRP314147. This data can be found here: [https://www.ncbi.nlm.nih.gov/sra/?term=SRP314147].
